# Impact of a one-year supervised physical activity program on long-term cancer-related fatigue and mediating effects of the gut microbiota in metastatic testicular cancer patients: protocol of the prospective multicentre, randomized controlled phase-III STARTER trial

**DOI:** 10.1186/s12885-024-11824-7

**Published:** 2024-01-15

**Authors:** Hwayoung Noh, Amélie Anota, Rodolf Mongondry, Renaud Meyrand, Carmen Dupuis, Camille Schiffler, Philippe Marijnen, Sabina Rinaldi, Joel Lachuer, Pekka Keski-Rahkonen, Marc J Gunter, Aude Fléchon, Béatrice Fervers, Olivia Pérol

**Affiliations:** 1 Departement of Prevention Cancer Environment, Léon Bérard Cancer Centre, Lyon, France; 2 INSERM U1296, Léon Bérard Cancer Centre, Lyon, France; 3 Nutrition and Metabolism Branch, International Agency for Research on Cancer (IARC-WHO), Lyon, France; 4 Direction of Clinical Research and Innovation, Léon Bérard Cancer Centre, Lyon, France; 5 INSERM U1052, Cancer Research Center of Lyon (CRCL), University Lyon 1, Lyon, France; 6 ProfileXpert, SFR santé Lyon-Est, CNRS UMR-S3453, INSERM US7, Lyon, France; 7 Department of Epidemiology and Biostatistics, School of Public Health, Imperial College London, W2 1PG London, UK; 8 Department of Medical Oncology, Léon Bérard Cancer Centre, Lyon, France

**Keywords:** Physical activity, Testicular cancer, Cancer-related fatigue, Health-related quality of life, Cancer-related sequelae, Gut microbiota, Gut microbiota-derived metabolites, Gut-brain axis

## Abstract

**Background:**

Testicular germ cell tumours (TGCTs) are the most common malignancy in men aged 15–40 years, with increasing incidence worldwide. About 33 ~ 50% of the patients present with metastatic disease at diagnosis. TGCT survivors experience short- and long-term sequelae, including cancer-related fatigue (CRF). Physical activity (PA) has established effects on reducing CRF and other sequelae and improving health-related quality of life (HRQoL). However, its impact on TGCT survivors has so far received little attention. The gut microbiota plays a crucial role in various physiological functions, including cognition and metabolism, and may mediate the effects of PA on CRF and other sequelae, but this has not been investigated in randomized controlled trials.

**Methods:**

This national, multicentre, phase-III trial will evaluate the impact of a one-year supervised PA program on CRF and other short- and long-term sequelae in metastatic TGCT patients receiving cisplatin-based chemotherapy combined with etoposide+/-bleomycin. It will also investigate potential mediating effects of the gut microbiota and its metabolites involved in the gut-brain axis on the relationship between PA and CRF and other sequelae. A total of 236 men ≥ 18 years of age with metastatic TGCT (seminoma and non-seminoma) will be enrolled before starting first-line chemotherapy in several French hospitals. The primary (CRF) and secondary (cognitive/psychological/metabolic sequelae, HRQoL, etc.) outcomes and gut microbiota and relevant metabolites will be assessed at inclusion, during and at the end of the one-year intervention, and annually until 10 years since inclusion to assess long-term sequelae, more specifically CRF, cardiovascular toxicities, and second primary cancer occurrence in this population.

**Discussion:**

This trial will provide comprehensive and novel insights into the effects of a long-term supervised PA program on CRF and other sequelae in metastatic TGCT patients receiving first-line chemotherapy. It will also contribute to understanding the potential role of the gut microbiota and its metabolites in mediating the effects of PA on these outcomes. The findings of this study will help the development of effective PA interventions to improve the health of TGCT survivors and may have implications for other cancer populations as well.

**Trial registration:**

The study was registered on ClinicalTrials.gov (NCT05588700) on 20 Oct. 2022.

## Background

Testicular germ cell tumours (TGCTs) are a relatively rare type of cancer but are the most common tumours in men aged 15–40 years [1]. Although TGCT remains a rare disease, the incidence has been increasing worldwide for 30 years, especially in Western countries [2]. TGCTs are divided into two histological types: seminoma (55–60%) or non-seminoma (40–45%). TGCT is associated with germ cell neoplasia in situ, a common precursor of seminoma and non-seminoma TGCT. Orchiectomy is the main treatment in TGCT. About one-third of patients with seminoma and half of patients with non-seminoma are metastatic at diagnosis [3, 4] and receive cisplatin-based chemotherapy (Bleomycin, Etoposide, and Cisplatin (BEP) or Etoposide and Cisplatin (EP)), administrated for 3 to 5 cycles. Some non-syringomatous TGCT patients also receive paclitaxel in addition to BEP [5, 6]. As TGCTs are highly sensitive to available treatments, the relative overall 10-year survival is > 95%, and most of these young patients can have a normal life expectancy after treatment [7–9].

Despite this good prognosis, TGCT survivors often suffer from short- and long-term sequelae, especially patients receiving chemotherapy [2]. The overall incidence rate of sequelae among TGCT survivors is 66.3 per 1,000 person-years [15]. Sequelae include cancer-related fatigue (CRF), a deteriorated health-related quality of life (HRQoL), cognitive impairment, and psychological/metabolic disorders [3–5]. In particular, CRF is one of the most common sequelae among cancer survivors [10] and is also frequently reported by TGCT patients and survivors. CRF may worsen and become chronic several years after treatment in TGCT patients [11]. Previous studies reported a prevalence of CRF of 15% after diagnosis, with an increase over time to 27% after a median follow-up of 19 years [12]. In addition to these self-reported outcomes, TGCT patients can experience other late adverse effects. Among them, cardiovascular diseases and second malignant neoplasms represent the most common potentially life-threatening late effects [13].

The modification of lifestyle factors including the practice of physical activity (PA) during oncological treatments has been identified consistently in the literature as a means to reduce CRF and other sequelae in cancer patients including TGCT [13, 14]. Numerous studies have shown the safety and benefits of PA performed concomitantly to treatments [15, 16]. PA improves body composition, reduces fatigue, and improves HRQoL [17–20]. The current body of evidence in exercise-oncology research is based on trials carried out predominantly in breast and prostate cancers. Yet, studies in patients with metastatic TGCT remain very rare [21]. Also, a reduced cancer-specific and all-cause mortality of post-diagnosis PA has been reported for 11 cancer sites but no data were available for TGCT survivors [22]. Multiple studies have highlighted the need to educate TGCT patients about practising a regular PA and its benefits on the reduction of fatigue, the improvement in HRQoL and psychological impairments, and its protective effect on cardiovascular diseases. Thus, PA could play a key role in reducing short- and long-term sequelae among TGCT patients and survivors [12, 13, 23–26]. PA is increasingly included in the standard oncology setting, and a growing body of evidence has outlined the beneficial potential of exercise to improve a broad range of physical/physiological and psycho-social endpoints [27, 28].

The human gut microbiota, a complex community of microorganisms dominated by bacteria, is an important modulator of host metabolic, immune, psychological, and cognitive functions [29]. Emerging evidence strongly links cancer treatment-related alteration in the gut microbiota and its metabolites [e.g. short-chain fatty acids (SCFAs), bile acids, and serotonin] involved in the gut-brain axis to CRF and other cognitive/psychological/metabolic sequelae of cancer survivors [30, 31]. Moreover, PA is a possible modulator of gut microbiota composition and diversity. Review papers [32, 33] with observational and intervention studies among non-cancer populations indicate that PA has positive effects on gut microbiota diversity accompanied by an increase in certain bacteria that produce beneficial metabolites such as bile acids, SCFAs, some neurotransmitters including gamma-aminobutyric acid (GABA) and serotonin. In addition, these recent data suggest that exercise-induced changes in gut microbiota could activate the hypothalamic-pituitary-adrenal (HPA) axis with reduced stress and improved psychological and cognitive function [33].

The excellent prognosis combined with the increasing incidence of TGCT has led to a growing number of TGCT survivors. Due to their younger age at diagnosis, the issue of short- and long-term sequelae is of particular concern in this population. Few PA programmes have been proposed for metastatic TGCT patients during cancer treatments. To our knowledge, the impact of PA on short- and long-term sequelae in TGCT survivors and the potential role of gut microbiota and its metabolites in the positive effects of PA on CRF have not been investigated in randomized controlled trials.

We will, therefore, conduct a national, multicentre, and phase III randomized controlled trial, STARTER (as**S**essmen**T** of a supervised physical **A**ctivity p**R**ogram on short- and long-term sequelae in patients with metastatic **TE**sticular germ cell tumou**R**) to evaluate the impact of a one-year supervised PA program on CRF and other sequelae in metastatic TGCT patients receiving chemotherapy. We will also evaluate potential effects of gut microbiota and its metabolites in the PA impact on CRF and other sequelae.

## Study hypotheses & objectives

### Study hypotheses

**Fig. 1:**
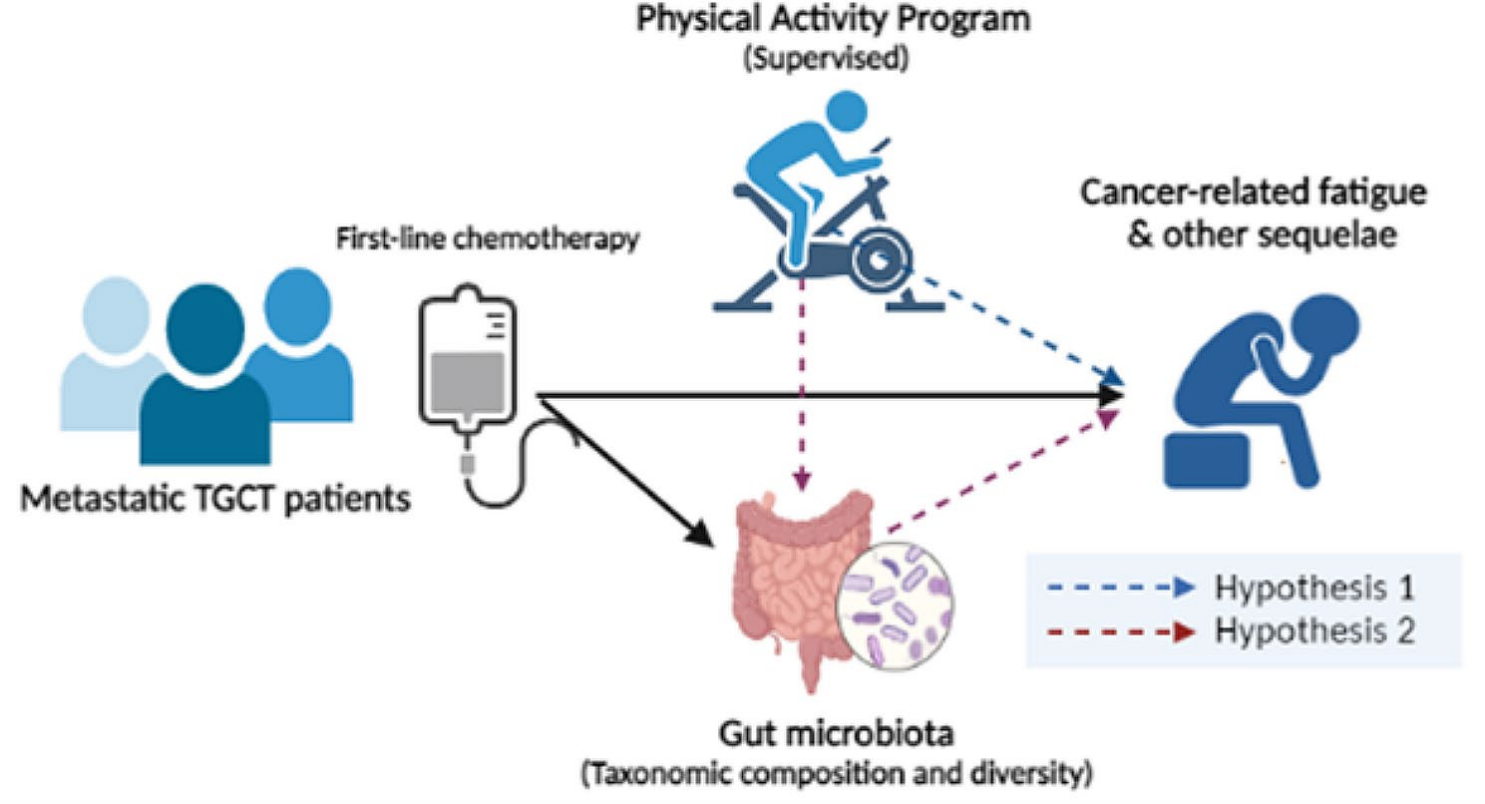
Hypotheses of the STARTER project

The study hypothesizes that [1] the practice of a one-year supervised PA program at the onset of first-line chemotherapy in patients with metastatic TGCT, reduces CRF and has positive effects on cognitive, psychological, clinical, and metabolic sequelae and their HRQoL and [2] the positive effects of PA on CRF and other sequelae in TGCT are mediated by the gut microbiota and its metabolites involved in the gut-brain axis (Fig. 1).

### Study objectives

Objective 1: to investigate the impact of a one-year supervised PA program implemented concomitantly with first-line cisplatin-based chemotherapy, on CRF (primary outcome, both physical and cognitive aspects of CRF at 3 years) and other short- and long-term sequelae (secondary outcomes: another dimension of CRF– emotional fatigue, HRQoL, cognition, anxiety/depression, anthropometry, physical condition, PA/sedentary behaviour level, pain and neuropathy, sustainable return to work, sleep quality, heart rate, TGCT relapse, cardiovascular toxicities, secondary primary malignancy, immune/inflammatory/liver function biomarkers) in first-line metastatic TGCT patients (Hypothesis 1).

Objective 2: to investigate how the gut microbiota (diversity and taxonomic composition) and its metabolites involved in the gut-brain axis (e.g., SCFAs, bile acids, GABA, tryptophan metabolites including serotonin) mediate the impact of PA on primary and secondary outcomes in metastatic TGCT patients receiving chemotherapy (Hypothesis 2).

## Study design/methods

The study protocol was approved by the French Ethics Committee for Personal Protection (CPP, reference number: 2022-A00542-41) and registered on *ClinicalTrials.gov* (NCT05588700). The study database was reported to the National Commission for Data Protection and Liberties (CNIL, reference number: 2016177).

### Study design

**Fig. 2:**
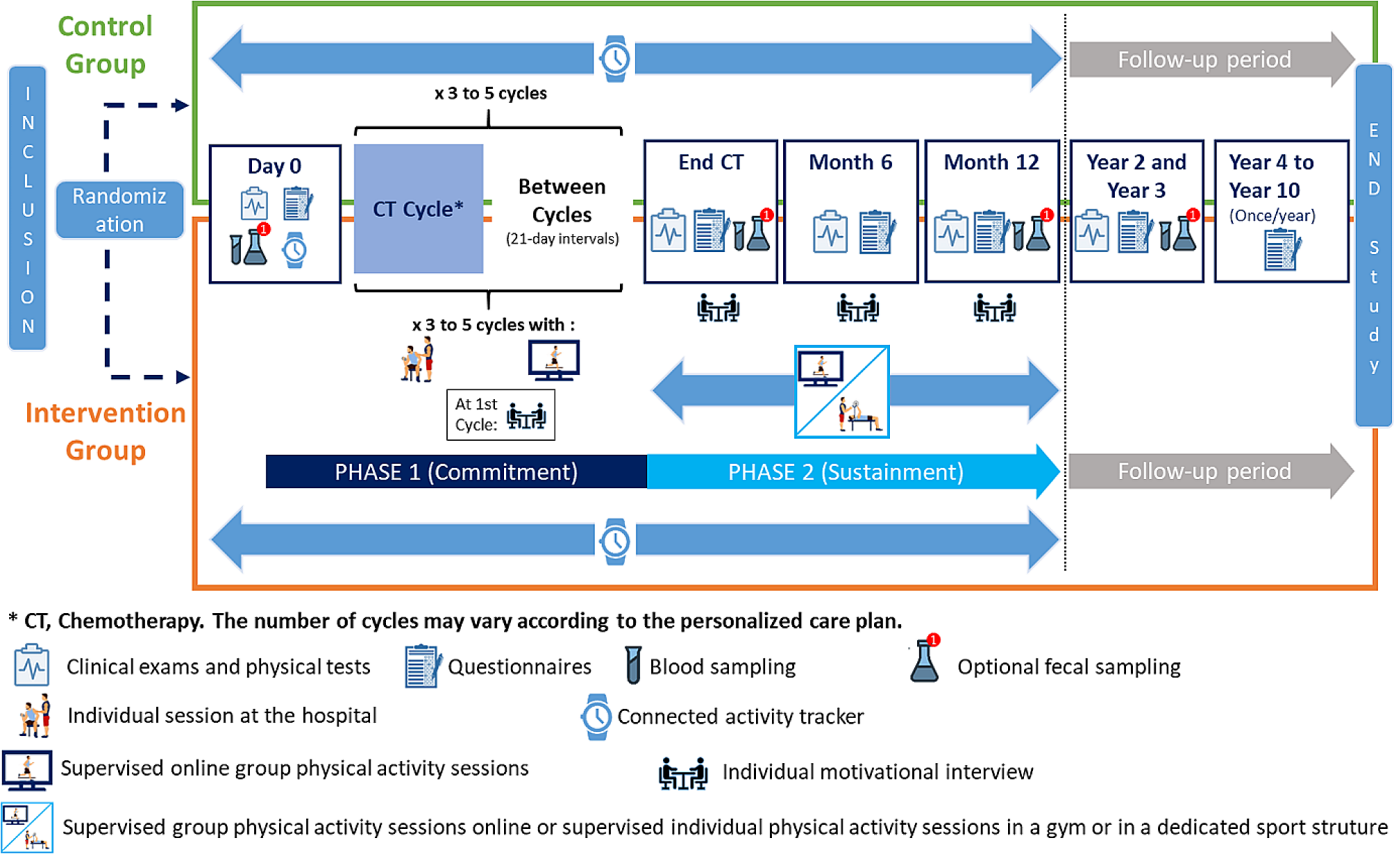
Study design of the STARTER trial

The STARTER study is a prospective, national multi-centre, open-label, two-armed, randomized controlled phase III trial (Fig. 2).

### Eligibility criteria

Inclusion criteria: Patients will have to meet all of the following criteria– (1) men ≥ 18 years of age, with a metastatic TGCT histologically confirmed (seminoma or non-seminoma), who have already undergone orchiectomy and will receive first-line cisplatin-based chemotherapy combined with etoposide +/- bleomycin; (2) who are willing and available to participate throughout the study; (3) who have the ability to practice PA, certified by an oncologist or a physician; (4) who have a smartphone, compatible with the “STARTER” application and having an internet connection (minimum version: Android 6 and iOS 10); (5) who are affiliated to a social security scheme; (6) who are able to read, write, and understand French.

Non-inclusion criteria: Patients will not be eligible if they have at least one of the following criteria– (1) localized TGCT; (2) symptomatic bone metastases; (3) symptomatic brain metastases, and/or central nervous system involvement with neurological deficits that prevent from walking; (4) history or co-existence of other primary cancer (except in situ cancer regardless of the site, basal cell carcinoma, and/or cancer in complete remission for more than 3 years); (5) any contraindication to PA or any condition precluding the practice of PA (e.g. uncontrolled hypertension, uncontrolled heart disease); (6) being unable to follow the study due to any medical, social, family, geographical, or psychological reasons throughout the study; (7) being deprived of liberty by judicial or administrative decision or protected by law; (8) concomitant participation to another PA trial; or (9) taking antibiotics in the previous two weeks prior to faecal sampling (only for the gut microbiota study).

### Recruitment

Participants will be recruited from several comprehensive cancer centres, hospitals, and clinics in France. After a systematic screening through patient files and/or the weekly multidisciplinary testicular cancer board, the study will be proposed to eligible patients by the investigator, with the support of a clinical research assistant. During the chemotherapy consultation, the investigators will check the eligibility criteria, deliver the study information sheet, and explain the objectives and the procedure to eligible patients. The patients will be given sufficient time for reflection, and the investigators will answer their questions if necessary. Patients who agree to participate will give informed consent, by means of a dated and signed informed consent form. A copy of the consent signed jointly by the investigator will be given to the patient. At the same time, the investigator, after a clinical examination, will issue a medical certificate of no contraindication to practice PA. The inclusion of patients is possible until the 3rd day of the first cycle of the first line of chemotherapy. The number of eligible patients who refuse to participate will be recorded in a screening dashboard.

### Randomization

At the first visit of the study (D0), patients will be randomly assigned (1:1 ratio) to one of the following arms: (1) Intervention arm - patients will receive PA recommendations at baseline, and a one-year PA program based on supervised sessions, motivational interviews and a connected activity tracker to wear during the whole intervention or (2) Control arm - patients will receive PA recommendations at baseline and a connected activity tracker to wear during one year.

Randomization will be stratified on (1) tumour classification: good vs. intermediate vs. poor prognosis [3, 4]; (2) PA level at inclusion assessed by the French National Observatory for PA and Sedentariness PA Questionnaire (ONAPS-PAQ), which is developed and validated to assess PA and sedentary behaviours in the French general population [34]: inactive vs. active vs. very active; (3) patient age: less than 40 years vs. 40 years and older.

An online randomization platform (Ennov Clinical Software) will be used to confirm the eligibility of the patient, indicate the criteria for stratification and proceed with randomization. The result of the randomization will be available immediately and a confirmation email will be automatically sent to the research staff of the investigating centre and the coordinating centre.

### Intervention

At baseline, participants in both arms will receive: (1) PA international recommendations for promoting health in the general population (i.e., 150 min of moderate PA/week), delivered orally by an adapted PA professional [35] and (2) a connected activity tracker to wear 24 h a day during the whole intervention (one year). All participants will be asked to a dedicated application that will be used to transfer the activity tracker data (i.e., the number of steps per day, heart rate, and sleep data) throughout the intervention. The user instructions for the activity tracker and mobile application will be given. The application will be also used to fulfil self-administered questionnaires by participants throughout the study.

PA intervention: Patients randomized in the intervention arm will additionally receive a one-year supervised PA program including 4-time individual motivational interviews.

Phase 1 (Commitment to PA)– during chemotherapy (~ 3 months): During the hospital stay for chemotherapy (5 consecutive days for each cycle– during 3 to 5 cycles), patients will be asked to perform 2 to 4 PA sessions of 30–60 min, supervised by an adapted PA professional. Sessions, performed at moderate intensity, will be individualized according to patient’s physical condition, fatigue level, and perceived exertion, and combine aerobic (ergometers) and resistance training (weight machines, resistance bands, free weights or bodyweight exercises) according to the possibilities of the different centres. Between chemotherapy cycles (21-day intervals), patients will be asked to perform 1 or 2 supervised, collective (max. 6 participants) live online PA sessions per week, proposed by a PA partner (Sporactio). Sessions of 30–60 min will be adapted according to the objective of each patient and their capacity and willingness of the day based on a discussion with an adapted PA professional at the beginning of each session. Activities combining aerobic and resistance training will be proposed to meet the patient’s objectives and adapted in a playful way to encourage investment and pleasure in practising. As far as possible, patients will be grouped in the same activities to encourage interaction between the participants, to stimulate their motivation and to facilitate the adaptation of the exercises according to their specific characteristics. An information meeting will be set up between professionals of the investigating centres and Sporactio to exchange on patient’s physical and clinical condition if needed.

Phase 2 (Sustainment of PA)– after chemotherapy (~ 9 months): Patients will be asked to perform 2 to 3 PA sessions per week at a moderate intensity. Patients will be offered 3 options for PA practice: supervised collective live online PA sessions (as in phase 1), sessions in partner fitness centres (in a “classic” environment with free access), or sessions in a dedicated sport structure, according to patient’s wish to practice a specific activity (support budget available for each patient). In all cases, communications between different PA professionals will take place to optimize PA programs for each patient.

In addition, during the entire duration of the program, patients in the intervention arm will benefit from four individual motivational interviews of 60 min each, conducted by a video conference and/or a phone call with a professional specialist in social-cognitive models including the transtheoretical model, developed by Prochaska and DiClemente [36], as well as the self-determination theory. The first motivational interview will take place between the first two cycles of chemotherapy to identify patient’s stage of behaviour change and to set the first objectives; the second motivational interview will be realized at the end of Phase 1 (end of chemotherapy) with the aim to accompany the patient to the next phase; the third one will take place 3 months after the start of Phase 2 (i.e., ~ 6 months after randomization) to ensure a follow up of the patient’s objectives, and the last motivational interview will take place at the end of the intervention (i.e., ~ 12 months) to make a balance sheet of the situation and to accompany the patient towards an autonomous PA practice.

Each motivational interview will be conducted according to the following process: (1) Engaging: creating a trusting and working relationship between the professional and the patient; (2) Focusing: establishing clear targets related to the interviews and an overall target related to the patient’s change process; (3) Evoking: collecting information from the patient about change developing his own motivations for change; (4) Planning: when the patient seems motivated and ready to make a change, getting the patient to commit and formulate a concrete plan of action for the change he wants to make (i.e., the patient formulates “what to change” and “how to change”).

### Evaluation modalities

Patients will undergo several assessments during the study: at inclusion (D0), at the end of chemotherapy (end CT, about 3 months, M3), at 6 months (M6), at the end of the study intervention (one year, M12), and then annually until 10 years (Y2 ~ Y10) to evaluate potential long-term sequelae. Assessments will include clinical examination, physical assessments, self-administered questionnaires and biospecimen collection and will be performed by a physician, an adapted PA professional, and a clinical research assistant when patients come in as part of their usual disease management.

At the first assessment, the clinical research assistant will explain the study modalities to the patient. All self-administered questionnaires will be completed by patients directly via the mobile application or website. They will receive notifications via the application or by email alerting them that a new questionnaire is available and has to be completed. If the patient does not complete the questionnaires, a reminder schedule will be provided (maximum 3 reminders). If the patient does not complete the questionnaire within the time limit, it will be automatically deleted. A system of alerts on the professional interface of the STARTER website will allow the clinical research assistant to follow the completion of the questionnaires and to contact patients if needed to limit missing data. The clinical research assistant will also be able to enter the data for the patient directly on the internet platform if the patient encounters difficulties in filling out the questionnaire. A messaging system on the STARTER application will be available to patients throughout the study to communicate with the research team about study progress and assessments. PA, heart rate and sleep data from the connected activity tracker will also be collected via the STARTER application.

### Data/biospecimen collection

The assessment details at each time point are described in Table 1.

#### Sociodemographic and clinical data

At inclusion, sociodemographic data including age, living situation, employment status, education, and socio-professional level will be collected by a self-administered questionnaire and will be filled out in an electronic case report form. Clinical data will be collected from the participant’s electronic medical record and will include diagnosis date, tumour histology, personal and familial history of TGCT, current treatment and sites of metastases.

#### Primary study outcome

*Cancer-related fatigue (CRF)*, considering both physical and cognitive fatigue scores at 3 years after the start of first-line chemotherapy [Time point: Y3]: CRF will be assessed by the European Organization for Research and Treatment of Cancer Quality of Life Questionnaire-FAtigue12 (EORTC QLQ-FA12) [37], a multidimensional instrument measuring CRF with 12 items including the evaluation of both physical and cognitive aspects.

#### Secondary study outcome

*Other dimensions of CRF* [Time points: D0, end CT (M3), M6, M12, then annually until Y10]: CRF will be assessed by the EORTC QLQ-FA12 [37]. Participants will be asked to rate their fatigue to a 4-point Likert scale ranging from “not at all” to “very much”. All the scores will be converted to a scale from 0 to 100, with higher scores indicating greater degrees of CRF.

*Health-related Quality of Life (HRQoL)* [Time points: D0, end CT (M3), M6, M12, and then annually until Y10]: HRQoL will be assessed by the EORTC QLQ (QLQ-C30), with 30 items categorized into five functioning scales (physical, role, cognitive, emotional, and social), a global QOL scale, three symptom scales (fatigue, nausea/vomiting, and pain) and six single symptom items (dyspnoea, insomnia, appetite loss, diarrhoea, constipation, and financial difficulties) [38, 39]. In addition, the testicular cancer-specific HRQoL will be assessed by EORTC QLQ-TC26 [40], with 26 items organised into 7 domains and 6 single items addressing: treatment side effects, treatment satisfaction, future perspective, work/education problems, physical limitations, infertility, family problems, sexual activity, sexual enjoyment, sexual problems, communication, body image problems and testicular implant satisfaction. Participants will be asked to respond to a Likert scale ranging from “not at all” to “very much” or from “very bad” to “excellent”. Subsequently, all the scores will be standardized on a scale of 0 to 100, with higher scores representing better functioning, better HRQoL and greater symptom burden.

*Cognition* [Time points: D0, end CT (M3), M12, Y2, and Y3]: Cancer-related cognitive disorders will be assessed by the Functional Assessment of Cancer Therapy-Cognitive Function (FACT-Cog) questionnaire [41]. The FACT-Cog is a validated self-administrated questionnaire to evaluate memory, attention, concentration, language, and thinking abilities. It consists of 37 items with four subscales: patients’ perceived cognitive impairments, perceived cognitive abilities, deficits observed or reported by others, and impact of cognitive changes on HRQoL. Participants will rate how often these situations occurred during the last seven days on a Likert scale ranging from “never” to “several times a day”, with higher scores indicating better perceived cognitive function.

*Anxiety/depression* [Time points: D0, end CT (M3), M6, M12, Y2, and Y3]: Anxiety/depression level will be assessed by the Hospital Anxiety and Depression Scale (HADS) questionnaire– Anxiety and Depression subscales [42] with 7 items for each with a scale of 0 to 3, allowing thus obtaining two scores (maximum score for each = 21).

*Anthropometric measurement* [Time points: D0, end CT(M3), M6, M12, Y2, and Y3]: Height (cm, at inclusion only) and body weight (kg) will be measured according to standardized procedures, and body mass index (BMI) will be calculated as body weight in kilograms divided by the square of height in meters (kg/m^2^). Body composition (e.g., body fat mass, muscle mass, etc.) will be assessed by bioelectrical impedance analysis.

*Physical activity (PA)/sedentary level* [Time points: D0, end CT (M3), M6, M12, and then annually untilY10]: PA and sedentary levels will be measured by the ONAPS-PAQ [34]. The questionnaire is designed to assess the level of PA and sedentary lifestyle during a typical week for the adult population with three parts and 21 questions: activities at work, travel for utilitarian purposes and leisure or home activities. This questionnaire also assesses physical inactivity at all times of life (travel, work, leisure). Additionally, the PA level will be assessed by combining the number of steps per day collected by the connected activity tracker [continuous measurement over the first year from D0 to M12].

*Physical condition and muscular strength* [Time points: D0, end CT (M3), M6, M12, Y2, Y3]: Cardio-respiratory endurance will be evaluated by the 6-minute walk test measuring walking distance covered in meters over 6 min in a 30-meter circuit, which is a validated test for cancer patients [43]. Muscular abilities (strength/endurance/muscular power) of the lower limbs will be evaluated by the sit-to-stand test, a simple, rapid and reliable method, scoring corresponding to the number of times the patient stood up in 30 s [44]. Prehensile muscular strength will be evaluated by the handgrip test with a validated hand dynamometer, measuring the maximum force exerted in kilograms by squeezing the handgrip as forcefully as possible for 5 s [45].

*Pain and neuropathies* [Time points: D0, end CT (M3), M6, M12, Y2, Y3]: Pain and neuropathy intensity will be determined using Visual Analogue Scales (VAS) with a scale of 0 to 10, indicating “no pain” to “worst pain” [46], and also evaluated by clinicians during planned clinical exams.

*Nutrition* [Time points: D0, end CT (M3), M6, M12, Y2, Y3]: Plant food, red/processed meat, fast/processed food, sweetened beverages, and alcohol intake, will be evaluated by a standardised scoring system developed to examine the adherence to the 2018 WCRF recommendations [47].

*Sustainable return to work* [Time points: M12, Y2, Y3]: Participants will be asked the number of consecutive working days without sick leave by a work status questionnaire.

*Motivation* [Time points: D0, end CT(M3), M12]: The motivation of participants to practice PA will be measured by a French questionnaire, PA Motivation Scale for Health (“Echelle de Motivation envers l’Activité Physique en contexte de Santé”, EMAPS) [48], covering 18 items of the following domains: intrinsic motivation, integrated regulation, identified regulation, introjected regulation, external regulation, and motivation. Participants will be asked to respond to a 7-point Likert scale ranging from “does not match at all” to “corresponds very strongly”.

*Sleep quality and heart rate* [Time points: continuous measurement over the first year from D0 to M12]: the data of participants’ sleep quality and heart rate will be collected continuously by the connected activity tracker during the period of the PA intervention for both arms.

*Tumour evaluation* [Time points: end CT (M3), M6, M12, and then annually until Y10]; TGCT relapse will be confirmed by CT scan/tumour-maker increase collected from the patient’s medical record.

*Cardiovascular toxicities and secondary primary malignancy* [Time points: M12, and then annually until Y10]: will be collected during conventional medical follow-up from the patient’s medical record.

*Biomarkers* [Time points: D0, end CT (M3), M12, and Y3]: Immune/inflammatory biomarkers (e.g., IL-1α/β, IL-6, IL-15, TNF-α) using validated methods; metabolic health (e.g., insulin, glucose, triacylglycerol, cholesterol), and liver function biomarkers (ALT/AST, bilirubin) will be measured from routine blood tests and study blood samples.

This study will also allow for the qualification of practice locations and characteristics of the living environment of measured objectively using spatial methods. For both groups, their satisfaction with the connected activity tracker will be evaluated [Time point: M12]. For the intervention group only, the aim will be to evaluate [Time point: M12]: (1) adherence to weekly PA sessions assessed by the number of sessions scheduled/performed during the different phases of the intervention; (2) satisfaction with the intervention assessed by a self-questionnaire; (3) association between the pre-intervention PA practice environment (type of activity, practice locations) and compliance with the intervention.

#### Gut microbiota study outcome

This ancillary study will be optional so that it does not hinder patient participation. Patients will be offered to participate in the ancillary study at baseline.

*Gut microbiota* [Time points: D0, end CT (M3), M12, and Y3]: Gut microbial diversity and taxonomic composition will be measured using full-length 16 S rRNA gene amplicon sequencing on DNA extracted from faecal samples [49]. Faecal samples will be taken using a FIT kit (OC-Auto Sampling tubes, Eiken, Japan) which includes all the necessary equipment for collecting. The first faecal sample will be taken in the hospital at the start of chemotherapy. The other samples will be taken by the patient at home. This kit will be given to the patient during the visits. Once the sample has been taken, the patient must indicate the date and time of the sample on a questionnaire and use a stamped envelope to send the sample and the questionnaire to the University of Lyon 1, responsible for storing and analysing the data. The mailing should be done as soon as possible, ideally within 24 h of the collection. The faecal sample received by post should be frozen and stored at -80 °C before NDA extraction. Total DNA will be extracted from the faecal samples using the PowerFecal DNA Isolation kit (MoBio Laboratories, Carlsbad, CA, USA). DNA extracts will be stored at -20 °C before use. The 16 S rRNA V1-V9 gene will be amplified and sequenced using Oxford nanopore technology (3rd generation sequencing) [49]. The sequence data will be processed bioinformatically using a pipeline within QIIME2 [50, 51] to calculate (1) the relative abundance (%Amplicon Sequence Variant, ASV) of bacterial taxa, (2) within-sample (alpha-) diversity using the phylogenetic tree indices of Chao1, Shannon and Faith phylogenetic tree indices, and (3) between-sample (beta-) diversity using Bray-Curtis and unweighted and weighted UniFrac distance matrices.

*Gut microbiota-derived metabolites* [Time points: D0, end CT (M3), M12, and Y3]: Metabolites involved in the gut-brain axis (e.g., SCFAs, bile acids, GABA, tryptophan metabolites including serotonin) will be measured in serum using targeted and untargeted metabolomic approaches [52, 53]. Blood samples will be taken at the investigating centres during patients’ visits (at inclusion, at the end of chemotherapy, at one year and at 3 years). Subjects will be asked to come in fasting for at least 8 h. Blood will be collected in two vacutainer SST tubes of 6 ml. The tubes containing the blood will be left at room temperature (and protected from light if possible) for one hour and then centrifuged at 30,000 rpm for 10 min at room temperature. The serum will then be aliquoted into tubes provided by the sponsor (500 µL in each tube). Serum aliquots will be stored at -80°c before use. First, SCFAs, bile acids, GABA, and tryptophan metabolites including serotonin will be measured in the blood (serum) using targeted metabolomics approaches: SCFAs by derivation of 1-(tert-butyldimethylsilyl) imidazole (MTBSTFA) and analysis by gas chromatography (GC) [52]; Bile acids, GABA and tryptophan metabolites including serotonin will be measured by liquid chromatography-tandem mass spectrometry (LC-MS/MS) in combination with the kit Biocrates MxP Quant 500 (Biocrates, Innsbruck, Austria). Other non-target microbial metabolites will be measured by ultra-high-performance liquid chromatography-quadrupole time-of-flight mass spectrometry (UHPLC-QTOF-MS).

*Medication and supplement intake* [Time points: D0, end CT (M3), M12, and Y3]: Drugs or supplements (e.g., antibiotics, metformin, proton pump inhibitors, laxatives, probiotics) [54, 55] that can interfere with the gut microbiota taken for the last one month will be collected by a questionnaire. In addition, patients taking antibiotics in the previous two weeks prior to faecal sampling will be excluded from the ancillary study.

**Table 1:** Summary of data/biological sample collection

Evaluation	Time points													
	Inclusion (D0)	End CT (M3)	M6	M12	Y2	Y3	Y4	Y5	Y6	Y7	Y8	Y9	Y10	
***Clinical Examination***														
Pain	X	X	X	X	X	X								
Tumour Evaluation		X	X	X	X	X	X	X	X	X	X	X	X	
Cardiovascular toxicities				X	X	X	X	X	X	X	X	X	X	
Second primary tumour				X	X	X	X	X	X	X	X	X	X	
***Questionnaires***														
CRF (QLQ-FA12)	X	X	X	X	X	X	X	X	X	X	X	X	X	
HRQoL (QLQ-C30/TC26)	X	X	X	X	X	X	X	X	X	X	X	X	X	
Cognition (FACT-Cog)	X	X		X	X	X								
Anxiety/depression (HADS)	X	X	X	X	X	X								
PA/sedentary levels (ONAPS-PAQ)	X	X	X	X	X	X	X	X	X	X	X	X	X	
Pain and neuropathies (VAS)	X	X	X	X	X	X								
Nutrition (WCRF)	X	X	X	X	X	X								
Sustainable return to work				X	X	X								
Motivation (EMAPS)	X	X		X										
Satisfaction with the connected activity tracker				X										
Satisfaction with the intervention				X										
Practice locations	X													
***Physical condition/fitness***														
6-min walk test	X	X	X	X	X	X								
Sit-to-stand test	X	X	X	X	X	X								
Handgrip test	X	X	X	X	X	X								
Anthropometrics	X	X	X	X	X	X								
Number of steps per day (connected activity tracker)	Every day, non-stop (up to M12)													
Heart rate (connected activity tracker)	Non-stop (up to M12)													
Sleep quality (connected activity tracker)	Non-stop (up to M12)													
***Gut microbiota study***														
Blood sampling	X	X		X		X								
Optional faecal sampling	X	X		X		X								
***Blood biomarkers***														
Gut microbial metabolites	X	X		X		X								
Inflammation/Immunity	X	X		X		X								
Liver function	X	X		X		X								

## Statistical considerations

### Sample size

In order to evaluate our primary endpoint and to detect a minimum significant difference ≥ 10 points in the CRF scores between the 2 groups at 3 years, with a standard deviation of 20, considering 2 dimensions of CRF (physical and cognitive fatigue) assessed by the EORTC QLQ-FA12 questionnaire, with a bilateral alpha, type I error of 0.025 (Bonferroni adjusted *p*-value for two targeted dimensions, overall risk of 5%), a statistical power of 90%, 200 patients with fatigue scores available at baseline and 3 years is required. Considering 15% of lost to follow-up or unavailable data and patients without fatigue scores available, 118 patients per group will be randomized (1:1), for a total of 236 patients.

### Analysis population

Statistical analyses will be performed on the Intent-to-Treat (ITT) population, including all randomized patients analysed according to the randomization scheme. A sub-population will be defined for the analysis of the primary endpoint, corresponding to a modified ITT population, i.e., all ITT patients with at least the available fatigue score at baseline and 3 years.

### Statistical analysis

All statistical analyses will be performed using the R statistical software (ver. 4.3.0, R Development Core Team, 2023) and SAS (ver. 9.4, The SAS Institute, Cary, NC, USA).

Categorical variables will be described using frequency distributions and percentages. The number of missing data will be indicated, but will not be taken into account for the calculations. Quantitative data will be described using the number of observations, mean, standard deviation, median, minimum and maximum values. The date of randomization will be considered as the reference date in all time calculations unless otherwise specified. An ITT analysis will be used to avoid attrition bias. Data at inclusion will be described in the ITT population and presented by the randomization arm. Results will be considered significant when the *p*-value is less than or equal to 0.05, except for the primary endpoint (alpha set at 0.025).

For the primary endpoint, the minimal important difference (MID) will be set to 10 points for each fatigue score (physical and cognitive) (MID = 0.5*SD) [56, 57]. These dimensions of the EORTC QLQ-FA12 will be analysed at 3 years. A Student-t test will be used to compare the scores between the two arms at 3 years, adjusting for the level of HRQoL at inclusion. In case of non-normal distribution, a non-parametric Mann-Whitney test will be performed. An analysis of the missing data profile at baseline and during follow-up will be performed to determine the missing data profile. An effort will be made to have all patients complete the baseline questionnaire as well as the 3-year questionnaire, to ensure the statistical power for the primary analysis. Stratified analyses will be performed according to stratification criteria of the randomisation.

### Decision rule

A single sufficient decision rule will be taken to interpret the results: the primary endpoint will be considered to be reached if at least one dimension among the two targeted ones (physical and cognitive fatigue) will be statistically significant in favour of the intervention arm, in absence of a statistically significant negative result on the other dimension.

For secondary endpoints, data at randomization will be compared between the 2 arms, and then the absolute and relative variations between inclusion and the different measurement times will be described and compared by arm. Longitudinal analysis of HRQoL will be performed by including all available measurement time until Y3. A mixed model for repeated measures will be performed including the randomized group, time, and intervention by time interaction, as well as random effect on intercept and time to reflect the individual deviance at baseline and over time. The proportion of patients with stable, deteriorated, and improved HRQoL using the MID at M12, and at Y3. Each HRQoL score from the EORTC questionnaire will be analysed. The MID will be fixed to 10 points for each HRQoL score of the EORTC questionnaires. Time to improvement or deterioration of HRQoL could also be proposed in complement using Kaplan-Meier survival estimation [58, 59]. A detailed statistical analysis plan will be written for each endpoint before the data is frozen.

For the gut microbiota study, to investigate whether the gut microbiota and its metabolites mediate the positive effect of PA on CRF and other short- and long-term sequelae in metastatic TGCT patients receiving chemotherapy, we will apply two steps of statistical analyses overall: Step 1 to test “the impact of PA on the chemotherapy-induced alterations in the gut microbiota and relevant metabolites” and Step 2 to test “the effect of the gut microbiota and relevant metabolites modulated by PA on CRF and other short- and long-term sequelae in metastatic TGCT patients receiving chemotherapy”.

To identify discriminated taxonomic features between the two arms at different taxonomic levels, we will apply the DESeq test (The R “DESeq” package) [60] corrected for multi-comparisons using the Benjamini–Hochberg procedure [61]. Then, we will conduct partial Spearman correlation analyses among identified taxonomic features, CRF and other sequelae scores, and levels of inflammation/immunity biomarkers. Prior to analysis, the taxonomic composition data will be centred log-ratio transformed for handling zeros in the dataset based on a Bayesian-multiplicative replacement according to a guideline for compositional analysis of omics-data [62].

To examine the difference in the diversity and metabolites of the gut microbiota between the two arms and its association with CRF and other sequelae scores and levels of inflammation/immunity, we will apply general linear models (GLMs) for α-diversity and gut microbial metabolites (e.g., SCFAs, bile acids, GABA, tryptophan metabolites) and permutational multivariate analysis of variance (PERMANOVA) and principal coordinate analysis (PCoA) for β-diversity.

In addition, to explore trajectories of composition, diversity and metabolites of the gut microbiota along with chemotherapy and PA intervention, we will use linear/non-linear mixed effect models (The R “TcGSA” package) [63], using the cubic function for time modelling.

All models applied in this study will be adjusted for factors associated with PA performance, the gut microbiota, and sequelae of TGCT patients using a Directed Acyclic Graph (e.g., age, BMI, body composition, other lifestyle factors– diet, smoking, alcohol intake, socioeconomic factors, initial psychological disorders, etc.).

## Discussion

Although recent evidence suggests TGCT patients practising a regular PA, reduce the prevalence of several adverse sequelae especially CRF, psychological disorders (e.g., anxiety and depression), cardiovascular toxicities, and second malignancies, and improve HRQoL [12, 13, 23–26], no prior studies have assessed the impact of a long-term supervised PA program in patients with metastatic TGCT concomitant to first-line chemotherapy, together with the mediating effects of gut microbiota and its metabolites on the PA impact.

An observational study assessed the association between the practice of moderate to vigorous PA and fatigue until 2 years after the start of treatment among cancer patients including TGCT and showed lower fatigue at each timepoint for patients who practiced one hour daily of PA [64]. In another observational study on the determinants of chronic fatigue in 812 TGCT survivors, being moderately and highly physically active had a protective effect on long-term fatigue (*p* <.001) [12]. An interventional study assessed a 12-week high-intensity interval training program among 63 TGCT survivors, including metastatic patients (8 (± 5.5) years after TGCT diagnosis) and reported significant improvement in fatigue and HRQoL with a mediation effect by cardiorespiratory fitness, and also on surrogate markers of mortality (i.e., multiple secondary outcomes including cardiovascular disease risk (*P* =.011), arterial thickness (*P* <.001), arterial stiffness (*P* <.001), post-exercise parasympathetic reactivation (*P* =.001), inflammation (*P* =.045)) [65, 66].

While overall PA is safe in cancer patients and cancer survivors [28], it is to be noted that another interventional study that aimed to evaluate the effect of high-intensity interval training during cisplatin-based chemotherapy for TGCT patients (median age 31 [21–50] years) reported an unexpectedly high number of thromboembolic events and the trial was stopped prematurely [67]. These results stress the importance of proposing an adapted PA program, taking into consideration the concomitant chemotherapy treatment with cisplatin, the patient’s physical condition, comorbidities, and preferences.

A feasibility study reported the desire of TGCT patients to be involved in PA programs with a preference for walking and low-intensity activities [68]. In addition, a study highlighted that TGCT survivors were more likely to engage in regular PA than age-matched healthy subjects [69]. Moreover, the duration of chemotherapy administration (five consecutive days at each cycle) offers a great opportunity to sensibilize patients deeply to the practice of PA and to support them to engage in regular practice; the access to PA sessions online between chemotherapy cycles will also reinforce patients’ commitment to PA (Phase 1 = “commitment to PA”). In addition, after chemotherapy, Phase 2 of the intervention, patients will be proposed several modalities according to their preference and physical/environmental condition (Phase 2 = “sustainment of PA”), which is more likely to maintain regular PA after the intervention and to observe higher impact on long-term sequelae. Concerning PA modalities, it has been reported that improvement in fatigue is greater when PA is initiated concomitant to cancer treatment [70]. Aerobic and mixed (aerobic and muscle strengthening) PA targeted at cardiorespiratory capacity development appears to be the most effective in reducing fatigue [70–72]. Dose-response analysis shows a maximum benefit in terms of fatigue reduction for moderate to intense PA compared to lower-intensity PA on the one hand [71, 73, 74], and high-intensity PA on the other hand [70, 75, 76]. The benefit appears to be greater when PA is performed in a supervised programme compared to an unsupervised one [20, 72, 77].The emergence of connected devices with activity trackers allows patients to practice a PA at their convenience and appears as a relevant alternative to traditional PA interventions. Connected devices allow PA interventions among a larger number of patients and overcome social and territorial inequalities. These devices propose an innovative way to remotely monitor patients according to their abilities and autonomy to promote widespread PA [78–80]. Our team evaluated the feasibility of using connected devices to practice PA in metastatic cancer patients [81]. A review on mobile health interventions to promote PA in cancer patients found that adding supervised sessions associated with the use of activity trackers was more effective in getting the patient to increase their PA level [82]. As the benefits of PA have been largely demonstrated in cancer patients, not proposing PA to patients during cancer treatments is ethically questionable [27, 28]. To take this into consideration with a control arm, activity trackers appear as a good opportunity to encourage patients to practice PA but with no direct and supervised intervention.

In addition to the PA intervention, the patient will be offered a complementary approach with MIs. This patient-centred approach aims to encourage patient engagement by focusing on his level of self-determination and changing his beliefs about the effects of PA (balance between perceived risks and benefits) through open-ended discussions. It has been shown that this type of technique facilitates a change in behaviour and makes it last over time [83–85]. It has been shown to positively impact motivation for PA and PA behaviour. Furthermore, a systematic review further suggested that motivational interview counselling was appropriate to change PA behaviours in cancer patients [86].

In addition, compelling evidence suggests that PA is one of the main environmental factors that can modulate gut microbiota diversity and composition. Previous studies in animal models [33] indicated that PA could affect the gut microbiota composition and diversity which might contribute to improving metabolic health. In an animal model [87], regular exercise was related to increases in overall diversity and butyrate-producing bacteria such as *Lactobacillus* along with an increase in butyrate (one of SCFAs) concentration in rats. Other animal studies in mice [88, 89] demonstrated that daily exercise might improve certain metabolic unhealthy states, such as diet-induced obesity and diabetes, by impacting the gut microbial composition. Previous human studies [33] showed similar trends as in animal studies with positive effects of PA on gut microbiota diversity and composition. A few cross-sectional observational studies [90, 91] in non-cancer populations showed that compared to sedentary populations, physically active populations (e.g. professional athletes) had higher gut microbial diversity and higher levels of certain bacterial taxa such as butyrate-producing bacteria (e.g. *Faecalibacterium prausnitzii* and *Roseburia hominis*) and obesity and metabolic health-related bacteria (e.g. *Akkermansia muciniphila*). A few prospective and randomized controlled trials [92, 93] with short-term (6 ~ 8 weeks) exercise programs in previously sedentary adults with higher BMI also demonstrated increased bacterial diversity and relative abundance of SCFA-producing bacteria after the intervention. Recently, emerging evidence strongly supported the key role of the gut microbiota in fatigue and other sequelae and the quality of life of cancer patients or survivors [30]. A previous cross-sectional study [31] among 88 patients with advanced, metastatic cancers showed that certain microbial taxa were associated with the fatigue of cancer patients - *Eubacterium hallii* was negatively associated with fatigue severity scores, whereas *Cosenzaea* was positively associated with fatigue scores. However, these previous studies had limitations to elucidate the causal relationships.

The STARTER trial, therefore, will provide novel insights into the impact of PA to reduce fatigue in TGCT survivors. This trial will also improve the knowledge of the benefits of a supervised PA program to reduce short- and long-term sequelae in this population. To date, only a few PA programmes have been proposed to TGCT patients during treatments, and the interventions were of short duration. Considering all the benefits of PA in cancer patients during treatments, we postulate that a one-year supervised PA program, in addition to MIs could help TGCT patients to counteract chemotherapy adverse effects and to facilitate the practice in autonomy and thus long-term maintenance. This trial will include an annual 10-year follow-up to assess long-term sequelae and more specifically fatigue, cardiovascular toxicities and second primary cancer in this population. The STARTER project will also increase the knowledge on the role of gut microbiota and its metabolites as a mediator in associations between PA and short- and long-term sequelae of TGCT survivors.

In conclusion, this trial will be the first long-term supervised PA program in TGCT patients receiving chemotherapy to understand the PA effects on CRF and other sequelae. It will allow us to better understand the mediating effects of gut microbiota and its metabolites on the PA-positive benefits on the adverse sequelae in the study population. The results of the STARTER trial will support the development of targeted PA guidelines to reduce CRF, improve HRQoL, and reduce other long-term sequelae in TGCT survivors, which will ultimately aim to improve their recovery and survival.

## Data Availability

No datasets were generated or analysed during the current study.

## Abbreviations

TGCT: Testicular Germ Cell Tumour
CRF: Cancer-Related Fatigue
HRQoL: Health-Related Quality of Life
PA: Physical Activity
SCFAs: Short-Chain Fatty Acids
GABA: Gamma-AminoButyric Acid
PAQ: Physical Activity Questionnaire
EORTC QLQ: The European Organisation for Research and Treatment of Cancer Quality of Life Questionnaire
FACT-Cog: Functional Assessment of Cancer Therapy-Cognitive Function questionnaire
HADS: Hospital Anxiety and Depression Scale
BMI: Body Mass Index
VAS: Visual Analogue Scales
ASV: Amplicon Sequence Variant
ITT: Intent-to-Treat
MID: Minimal Important Difference
GLM: General linear models
